# Epidemiological and clinical characteristics of community‐acquired and nosocomial influenza cases and risk factors associated with complications: A four season analysis of all adult patients admitted in a tertiary hospital

**DOI:** 10.1111/irv.12823

**Published:** 2020-10-30

**Authors:** Maria Isabel Fullana Barceló, Javier Asensio Rodriguez, Francisca Artigues Serra, Adrian Ferre Beltran, Pilar Salva D’agosto, Maria Almodovar Garcia, Maria del Carmen Lopez Bilbao, Pilar Sanchis Cortés, Jorge Reina Prieto, Melchor Riera Jaume

**Affiliations:** ^1^ Internal Medicine Department Hospital Universitario Son Espases Palma de Mallorca Spain; ^2^ Internal Medicine Service Hospital de Inca Inca Balearic Islands Spain; ^3^ Preventive Medicine Service Hospital Universitario Son Espases Palma de Mallorca Spain; ^4^ Department of Chemistry University of Balearic Islands Palma de Mallorca Spain; ^5^ Virology Unit Clinical Microbiology Service Hospital Universitario Son Espases Palma de Mallorca Spain; ^6^ Infectious Diseases Section Internal Medicine Department Infectious Diseases and HIV Group Hospital Universitario Son Espases IDISBA Palma de Mallorca Spain

**Keywords:** community‐acquired influenza, complications, epidemiology, hospital‐acquired influenza

## Abstract

**Background:**

Information on the characteristics of patients with nosocomial influenza and associated complications is scarce. This study compared epidemiological and clinical characteristics of patients admitted with hospital‐acquired influenza (HAI) to those with community‐acquired influenza (CAI) and analyzed risk factors associated with complications.

**Methods:**

This retrospective, observational study included all adult patients with confirmed influenza virus infection admitted to Son Espases University Hospital during the influenza season in Spain (October to May) from 2012‐2013 to 2015‐2016. Symptom onset before admission was included as CAI, and 2 days after admission or within 48 hours after previous discharge were considered as HAI.

**Results:**

Overall, 666 patients with laboratory‐confirmed influenza were included; 590 (88.6%) and 76 (11.4%) had CAI and HAI, respectively. Baseline characteristics and vaccination rates were similar in both groups. Patients with HAI had significantly fewer symptoms, less radiological alterations, and earlier microbiological diagnosis than those with CAI. Eighty‐five (14.4%) and 20 (27.6%) CAI and HAI patients, respectively, experienced at least one complication, including septic shock, admission to the intensive care unit, mechanical ventilation or evolution to death (any one, *P* = .003). Univariate and multivariate binary logistic regression was performed to assess independent risk factors associated with the occurrence of complications: nosocomial infection, diabetes, oseltamivir treatment, having received no vaccination, microbiological delay, dyspnea, and the state of confusion were the most important significant factors.

**Conclusions:**

Our study shows the need to implement microbiological diagnostic measures in the first 48 hours to reduce HAI frequency and associated complications.

## 1 INTRODUCTION

The influenza virus contributes significantly to the number of hospital admissions during the annual outbreak months. Among the existing viral pathogens, influenza remains the most clinically significant viral cause of community‐acquired pneumonia in adults, and it is a key cause of hospital‐acquired pneumonia.^1^ However, the impact of each influenza epidemic on morbidity and mortality varies considerably depending on the prevalence of the influenza virus in each season. Since 2009, following the new influenza A (H1N1) pdm09 virus pandemic, the use of rapid polymerase chain reaction (PCR) tests on respiratory samples has been extended in hospitals.^2^ Outbreaks of nosocomial infection usually occur during the seasons of community infection, predominantly in units with hematological and immunosuppressed patients.^3^, ^4^, ^5^, ^6^ Comorbidities such as chronic bronchitis, immunosuppression,^7^ diabetes, chronic kidney disease, nosocomial infection,^8^ and malnutrition^9^ have been previously described as a risk factors associated with complications like septic shock, need for mechanical ventilation, admission in intensive care unit (ICU) and death. However, only few studies have been conducted on the sporadic nosocomial cases of influenza and their associated complications, with complete clinical and virologic data.^10^, ^11^ Identifying and understanding risk factors of hospital‐acquired influenza (HAI) infection is important to implement control measures to reduce morbidity and mortality.

In Spain, the epidemiological surveillance of influenza is conducted through sentinel networks that are integrated into the influenza surveillance system (ScVGE). Surveillance on cases of confirmed influenza requiring hospitalization has been conducted since 2010; however, the data for all patients admitted for influenza have not been systematically collected. The main strategy for the prevention and control of influenza in hospitals is providing vaccination to people at risk and healthcare workers. This is, coupled with precautions against transmission through infected droplets, and early treatment with antivirals for people admitted for influenza.^12^, ^13^ However, adoption of these measures is highly variable between hospitals. A recently published study showed a reduction in nosocomial infection and length of hospital stay after the application of a real‐time surveillance system.^14^ This study therefore aimed to compare the epidemiological and clinical characteristics of patients who required hospital admission and diagnosed with community‐acquired influenza (CAI) and HAI or nosocomial. Additionally, we aimed to determine risk factors associated with complications and analyze whether nosocomial infection is also a risk factor for the development of complications. The secondary aim was to compare the rate of nosocomial infection in different seasons.

## 2 METHODS

### 2.1 Design, study population and definitions

This was a retrospective, observational study of adult patients with influenza virus infection admitted to Son Espases University Hospital (HUSE) from the influenza seasons 2012‐2013 to 2015‐2016. The period of influenza surveillance in Spain ranges from 40 epidemiological weeks of 1 year up to 20 weeks of the following year (October to May).^15^ All adult patients (aged > 18 years) with laboratory‐confirmed influenza virus infection were included. A case of confirmed influenza was defined as adult patients admitted for influenza syndrome (onset of systemic symptoms such as fever, malaise, myalgia, and respiratory symptoms including cough, sore throat shortness of breath, and absence of other suspected diagnosis) confirmed by PCR of ribonucleic acid detection in respiratory samples processed in the microbiology laboratory of Son Espases University Hospital. CAI cases were defined as cases in patients in whom symptom onset occurred before admission, without any previous hospitalization within 48 hours. HAI cases were defined as cases in patients with symptoms onset 2 days after admission, when the admission was for reasons other than respiratory symptoms, or patients who had new onset of symptoms within 48 hours after discharge and hence required a new hospital admission. Exclusion criteria were not applicable for our study because we included all individuals regardless of whether they had (prior to admission) or did not have (acquired after admission) influenza.

### 2.2 Data collection

The medical charts for positive cases at the microbiological department of HUSE were reviewed. Data were extracted from the medical charts using a case report form; the data collected included the date of admission and discharge, date of any previous discharges, signs and symptoms related to flu, date of symptoms onset and diagnosis, epidemiological variables, comorbidities, vaccination status for the current influenza season, analytical and radiological findings used to make a diagnosis, treatment with antiviral and antibiotics, and complications during the admission.

### 2.3 Procedures and settings

Detection of influenza viruses was performed using a commercial real‐time PCR (RT‐PCR: Allplex Respiratory Full Panel Assay; Seegen) which enables simultaneous and differential detection of 16 respiratory viruses, including influenza A and B and subtypes H1N1 and H3N2. It was performed 5 days a week during the influenza season. HUSE is an 800‐bed tertiary care center and is the reference hospital for the follow‐up of serious influenza cases in the Balearic Islands. An infection prevention policy is followed in the hospital, and respiratory hygiene precautions are taken for people treated for respiratory symptoms in the emergency department. Additionally, respiratory sampling was performed (oropharyngeal smear or other respiratory samples) in people admitted for suspected influenza. Up until 2018, only confirmed cases were isolated. Based on the hospital protocol, patients with confirmed influenza or patients with influenza‐like illness with comorbidities (immunosuppression, heart disease, lung disease or chronic kidney disease) were treated with oseltamivir.

### 2.4 Description of complications

Complications associated with flu included septic shock, admission to the ICU, need for mechanical ventilation and death. Other complications, less commonly described as associated with influenza, are described in the results section of this paper.

### 2.5 Statistical analysis

Numerical variables are expressed as medians and interquartile ranges (IQR) and categorical variables as frequencies and proportions. Differences between CAI and HAI were analyzed using the Mann‐Whitney *U* tests for continuous variables and chi‐square tests or Fisher's exact tests for categorical variables. Univariate binary logistic regression was performed to assess the risk factors of nosocomial infection associated to complications. The factors investigated in the univariate analysis were proven as risk factors in previous studies. Those with a *P*‐value <.1 in the univariate analysis were included in the multivariate binary logistic regression model to adjust for the effect of confounding factors on complications. A stepwise backward method was used in the final multivariate model to ascertain odds ratios (OR) and coinciding 95% confidence intervals (CI). Two‐tailed *P*‐values of less than .05 were considered to indicate statistical significance. Statistical analyses were performed using SPSS version 23.0 (SPSS Inc).

## 3 RESULTS

**Figure 1:**
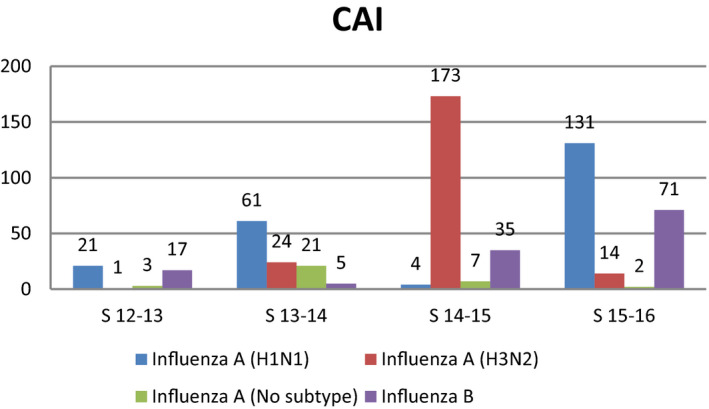
Distribution of community‐acquired influenza cases in each season based on the flu subtype. At the bars are indicated the cases in absolute numbers. CAI, Community‐acquired influenza; S, season

**Figure 2:**
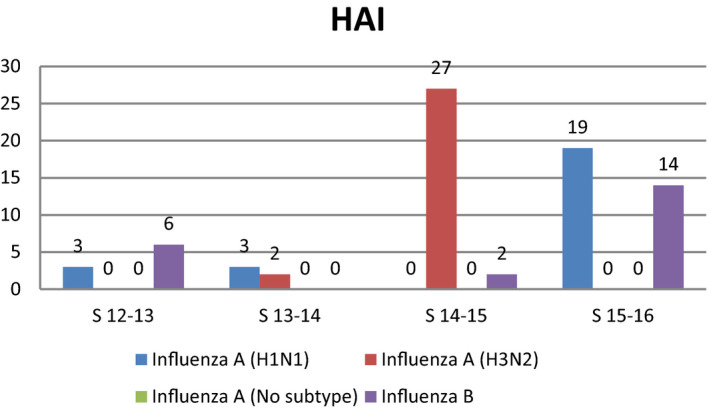
Distribution of hospital‐acquired influenza cases in each season based on the flu subtype. At the bars are indicated the cases in absolute numbers. HAI, Hospital‐acquired influenza; S, season

**Figure 3:**
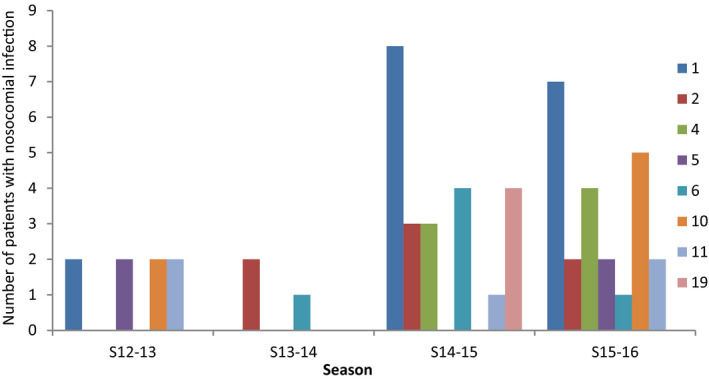
Distribution of nosocomial cases in the most frequent departments based on season. The *x*‐axis shows the number of the department and the *y*‐axis displays the absolute number of patients with nosocomial infection. S: season; 1: Internal medicine; 2: Pneumology; 4: Cardiology; 5: Intensive medicine (ICU); 6: Hematology; 10: Traumatology; 11: Cardiac surgery; 19: Psychiatry

Overall 666 patients with laboratory‐confirmed influenza were included over the four seasons; 590 (88.6%) and 76 (11.4%) patients were diagnosed with CAI and HAI, respectively. The distribution of the subtypes by seasons and the rate of nosocomial cases are shown in Figures 1,2, and Table 1. More nosocomial cases were diagnosed in the last seasons of the study with an incidence of 12% (*P* = .036). No differences in the rate of HAI were observed based on subtype when subtypes A and B were compared. Median time from hospital admission until HAI diagnosis was 10 days (IQR 5‐14), and median time between respiratory symptom onset and microbiological diagnosis was 1 vs 4 days for those with HAI and CAI, respectively (*P* < .0001). Distribution of the diagnosis of nosocomial cases based on department and season is shown in Figure 3. Most nosocomial cases were diagnosed in the internal medicine department (n = 8 in 2014‐2015 and n = 9 in 2015‐2016), followed by the traumatology, hematology, and cardiology departments.

**Table 1:** Clinical and demographic characteristics of community‐ and hospital‐acquired influenza patients

Clinical characteristics	Community‐acquired influenza n = 590	Hospital‐acquired influenza n = 76	*P*‐value
Age, median (IQR)	67.0 (53.0‐77.0)	66.5 (51.0‐77.8)	
Sex, (female), n (%)	288 (48.8)	40 (52.6)	
Smoking, n (%)	178 (30.2)	20 (26.3)	
COPD, n (%)	148 (25.1)	12 (15.8)	
Pregnancy, n (%)	5 (0.8)	0 (0.0)	
Obesity, n (%)	82 (13.9)	11 (14.5)	
Heart disease, n (%)	192 (32.5)	30 (39.5)	
Chronic kidney disease, n (%)	75 (12.7)	9 (11.8)	
Diabetes, n (%)	173 (29.3)	20 (26.3)	
Immunosuppression, n (%)	120 (20.3)	16 (21.1)	
HIV, n (%)	41 (6.9)	4 (5.3)	
Malignancy, n (%)	36 (6.1)	6 (7.9)	
Corticotherapy, n (%)	16 (2.7)	2 (2.6)	
Others, n (%)	27 (4.6)	4 (5.3)	
Vaccinated, n (%)	231 (39.2)	27 (35.5)	
Interval from symptoms to diagnosis (days), median (IQR), n total	4.0 (2.0‐7.0), 589	1.0 (0.0‐4.0), 76	.000
Oseltamivir, n (%)	323 (54.7%)	37 (48.7%)	
Antibiotics, n (%)	557 (94.4%)	60 (78.9%)	.000
Season, n (% in the same season)			
T12‐13, N = 51	42 (82%)	9 (18%)	.036
T13‐14, N = 116	111 (96%)	5 (4%)	
T14‐15, N = 248	219 (88%)	29 (12%)	
T15‐16, N = 251	218 (87%)	33 (13%)	
Subtype, n (%)			
Influenza A (H1N1)	217 (36.8%)	25 (32.9%)	
Influenza A (H3N2)	212 (35.9%)	29 (38.2%)	
Influenza A no subtype	33 (5.6%)	0 (0.0%)	
Influenza B	128 (21.7%)	22 (28.9%)	

Abbreviations: COPD, chronic obstructive pulmonary disease; IQR, interquartile range; S, season.

### 3.1 Demographic characteristics and comorbidities

Upon comparison of patients with HAI and CAI, no differences were observed in mean age, sex, or in the main comorbidities, including immunosuppression (Table 1). A small proportion of chronic obstructive pulmonary disease patients were diagnosed with HAI, and there were no HAI cases among pregnant women. Finally, there was no difference in the proportion of patients who received the influenza vaccination in the CAI and HAI groups (39% vs 35%, *P* = .541).

### 3.2 Clinical characteristics and initial treatment

Patients with HAI presented with cough, myalgia, and a breathless sensation less frequently, but a higher proportion of patients experienced confusional symptoms (15.8% vs 8.8%, *P* = .052) (Table 2). Upon physical examination, we did not observe differences in the heart or respiratory rates; however, patients with HAI had lower systolic blood pressure. As for laboratory parameters, it was noted that patients with HAI had lower hematocrit and albumin levels. Patients with HAI less frequently presented with the radiological criteria of pneumonia than those in the CAI group (13.2%, vs 29.7%, *P* = .003). Additionally, they had higher levels of oxygen saturation (96% vs 93%, *P* < .000) and other altered respiratory parameters such as lower Kirby index (PaO_2_/ FiO_2_) (257 vs 276 mmHg, *P* = .374) and a greater alveolar‐arterial gradient adjusted for age (18.9 vs 17.4, *P* = .700). With the exception of a bacterial co‐infection that occurred in 9.2% and 7.9% of those with HAI and CAI, respectively, there were no significant differences in the rates of co‐infection with other viruses between the groups. Antiviral treatment (oseltamivir) was given in 49% and 55% of HAI and CAI patients (*P* = .318), while 79% and 94% of patients, respectively, received antibiotics (*P* = .000).

**Table 2:** Symptoms, signs, and main analytical parameters in community‐ and hospital‐acquired influenza patients

Clinical characteristics	Community‐acquired influenza n = 590	Hospital‐acquired influenza n = 76	*P*‐value
Cough, n (%)	515 (87.3%)	58 (76.3%)	.009
Fever, n (%)	416 (70.5%)	60 (78.9%)	
Myalgia, n (%)	157 (26.6%)	9 (11.8%)	.005
Dyspnea, n (%)	406 (68.8%)	40 (52.6%)	.005
State of confusion, n (%)	52 (8.8%)	12 (15.8%)	.052
Heart rate, median (IQR), n total	96.0 (82.5‐109.5), 577	91.0 (80.0‐104.0), 73	
Systolic blood pressure [mmHg], median (IQR), n total	132.0 (114.0‐148.0), 582	122.0 (110.0‐138.5), 73	.017
Respiratory frequency, median (IQR), n	26.0 (20.0‐32.0), 168	24.0 (18.0‐33.8), 8	
Oxygen saturation [%], median (IQR), n	93.0 (90.0‐95.0), 579	96.0 (93.0‐98.0), 76	.000
Oxygen saturation <93%, n/n (%)	245/579 (42.3%)	17/76 (22.4%)	.001
PaO_2_/FiO_2_, median (IQR), n	276.2 (242.9‐309.5), 500	257.1 (211.4‐338.1), 27	
Alveolar‐arterial gradient, median (IQR), n	46.0 (38.1‐54.8), 517	44.2 (31.2‐86.4), 27	.700
Alveolar‐arterial gradient adjusted for age, median (IQR), n	17.4 (14.3‐19.5), 547	18.9 (14.8‐24.2), 48	.005
Lymphocyte [×10^9^/L] median (IQR), n	980.0 (670.0‐1437.5), 588	1160.0 (702.5‐1507.5), 76	
Hematocrit [%], median (IQR), n	39.7 (35.3‐43.0), 589	33.1 (29.3‐39.0), 75	.000
Platelet [×10^9^/L], median (IQR)	196.0 (150.0‐252.0)	227.5 (163.7‐326.2)	.027
Creatinine [mg/dL], median (IQR), n	0.9 (0.7‐1.1), 589	0.8 (0.7‐1.1), 76	
Urea [mg/dL], median (IQR), n	36.0 (28.0‐52.0), 584	39.0 (29.0‐59.0), 75	
Albumin [mg/dL], median (IQR), n	34.7 (31.4‐37.5), 423	32.1 (28.0‐35.2), 51	.000
C‐reactive protein [mg/dL], median (IQR), n	5.1 (2.0‐11.9), 456	6.1 (1.9‐13.3), 54	
Radiological infiltrate, n (%)	175 (29.7%)	10 (13.2%)	.003

Abbreviations: IQR, interquartile range; PaO_2_/FiO_2_, partial pressure of oxygen/ fraction of inspired oxygen.

### 3.3 Course of illness and complications

Overall, 85 (14.4%) and 20 (27.6%) patients in the CAI and HAI groups experienced at least one complication, respectively (*P* = .003, Table 3). In total, 39 patients died, 5.3% and 10.5% of whom were in the CAI and HAI groups, respectively (*P* = .072). ICU admission was required in 9.5% of CAI patients and 22.4% of HAI patients. Five patients with CAI presented with other complications: myopericarditis (n = 1), rhabdomyolysis (n = 2), Guillain‐Barre syndrome (n = 1) and meningoencephalitis (n = 1).

**Table 3:** Complications and prediction scales in community‐ and hospital‐acquired influenza patients

Complications	Community‐acquired influenza n = 590	Hospital‐acquired influenza n = 76	*P*‐value
At least one complication, n (%)	85 (14.4%)	21 (27.6%)	.003
Septic shock, n (%)	40 (6.8%)	10 (13.2%)	.047
Mechanical ventilation, n (%)	53 (9.0%)	14 (18.4%)	.010
Intensive care unit admission, n (%)	56 (9.5%)	17 (22.4%)	.001
Death, n (%)	31 (5.3%)	8 (10.5%)	.072

### 3.4 Risk factors associated with influenza complications

In univariate analysis, pregnancy, obesity, diabetes, interval between symptoms and microbiological diagnosis, oseltamivir treatment, not having been vaccinated, nosocomial infection, lower oxygen saturation, and symptoms of dyspnea and confusion were significantly associated with complications (Table 4). After adjustment for age, sex, and comorbidities, these factors remained significantly associated with complications. In multivariate analysis, nosocomial infection (OR [95% CI] = 3.39 [1.72‐6.67]), diabetes (OR [95% CI] = 1.67 [1.00‐2.79]), oseltamivir treatment (OR [95% CI] = 1.67 [1.02‐2.72]), no vaccination (OR [95% CI] = 0.54 [0.32‐0.93]), interval between symptoms and microbiological diagnosis (OR [95% CI] = 1.07 [1.03‐1.12]), dyspnea (OR [95% CI] = 2.10 [1.17‐3.77]) and state of confusion (OR [95% CI] = 6.07 [3.22‐11.46]) were independent factors significantly associated with an increased frequency of complications after adjustment for age and sex. Vaccination conferred protection against complications (OR [95% CI] = 54[0.32‐0.93]) and pregnancy appeared as an independent factor significantly associated with complications, but a small number of pregnant patients were included.

**Table 4:** Univariate and multivariate binary logistic regression analyses of independent factors significant for complications associated with influenza

Values	No complication (N = 560)	Complication (N = 106)	*P*‐value	Crude OR	95% CI)	*P*‐value	Adjusted OR	95% CI^a^	*P*‐value
Age, median (IQR)	67 (52‐78)	65 (54‐75)	.475	1.00	0.99‐1.01		1.00	0.98‐1.02	
Sex (female), n (%)	276 (49)	52 (49)	.965	0.99	0.65‐1.50		1.03	0.62‐1.69	
Pregnancy, n (%)	2 (0.35)	3 (2.8)	.031	8.13	1.34‐49.23	.023	9.88	1.40‐69.79	.022
Smoking, n (%)	159 (28)	39 (37)	.083	1.47	0.95‐2.27		1.40	0.80‐2.44	
COPD, n (%)	136 (24)	24 (23)	.716	0.91	0.56‐1.50		0.66	0.36‐1.22	
Obesity, n (%)	71 (13)	22 (21)	.033	1.80	1.06‐3.07	.030	1.19	0.64‐2.21	
Heart disease, n (%)	178 (32)	43 (40)	.197	1.45	0.95‐2.23		1.30	0.76‐2.22	
Chronic renal failure, n (%)	67 (12)	17(16)	.274	1.41	0.79‐2.51		1.54	0.77‐3.07	
Diabetes, n (%)	153 (27)	39 (36)	.132	1.53	0.99‐2.37	.054	1.67	1.00‐2.79	.052
Immunosuppression, n (%)	116 (21)	20 (19)	.965	0.89	0.53‐1.51		0.88	0.48‐1.63	
Interval from symptoms to diagnosis., median (days), (IQR)	5.13 (2‐6)	6.32 (2.75‐8.25)	.047	1.04	1.01‐1.08	.024	1.07	1.03‐1.12	.000
Oseltamivir, n (%)	281 (50)	70 (66)	.007	1.81	1.17‐2.80	.007	1.67	1.02‐2.72	.041
Antibiotics, n (%)	518 (92)	99 (93)	<.000	1.15	0.50‐2.63		0.83	0.32‐2.12	
Vaccinated,n (%)	228 (40)	30 (28)	.016	0.57	0.36‐0.91	.017	0.54	0.32‐0.93	.025
Nosocomial infection, n (%)	55 (10)	21 (20)	.003	2.27	1.31‐3.94	.004	3.39	1.72‐6.67	.000
Dyspnea, n (%)	364 (65)	82 (77)	.013	1.84	1.13‐2.99	.014	2.10	1.17‐3.77	.013
State of confusion, n (%)	36 (6)	28 (26)	<.000	5.23	3.02‐9.04	.000	6.07	3.22‐11.46	.000
Sat. O_2_ ^b^, median (IQR)	94 (91‐96)	93(87‐96)	.064	1.59	1.04‐2.42	.033	1.52	0.93‐2.48	

Abbreviations: CI, Confidence interval; IQR, Interquartile range; OD, Odds Ratio.

^a^ Multivariate analysis.

^b^ Oxygen saturation lower than 93%.

## 4 DISCUSSION

In this study, which was conducted spanning four consecutive influenza seasons, the rate of HAI was 11.4%. Unlike other studies,^10^, ^11^, ^16^, ^17^ we did not observe significant differences in age, baseline comorbidities and rates of treatment with oseltamivir between patients with HAI and CAI. Patients with HAI were diagnosed earlier and had fewer respiratory symptoms and radiological criteria for pneumonia; however, they more frequently presented with major complications (death and ICU admission). In multivariate analysis after adjustment for age and sex, HAI acquisition was associated with experiencing complications (OR [95% CI] = 3.39 [1.7‐6.67]).

During the four seasons investigated, we did not observe differences in the subtypes of influenza viruses that caused CAI and HAI. The proportion of patients who acquire influenza in the hospital varies in literature. This ranges from 9.6^17^ to 23.6%^11^ up to 35%^4^ in studies conducted in a single hospital during one influenza period, to 4.3%^18^ or 5.6%^10^ in multicenter studies with more than one influenza period of observation in Australia and Spain, respectively. The rate of 11.4% observed in our study is similar to that reported by previous authors in Spain.^6^, ^19^ The discrepancy in the previously reported rates may be due to variations in study design differing definitions for HAI, differences in influenza periods investigated, or RT‐PCR used for diagnosis. In the lack of a standardized definition for HAI, our definition was derived from the incubation time of the influenza A and B,^20^ considering the admission date and a previous discharge. In our study mortality in HAI was 10.5%, similar to other studies, which ranges from 7% to 18%.^10^, ^11^, ^16^, ^17^ Thus far, emphasis has been placed on nosocomial bacterial infections, which are more frequent than viral infections.^21^ With the ongoing coronavirus disease (COVID‐19) pandemic, this may change, as it will be imperative to conduct RT‐PCR investigations for respiratory viruses and coronaviruses in patients at admission, or in patients admitted for pneumonia or who develop symptoms during their hospital stay.^22^, ^23^ Unlike other studies, there were no differences in age or comorbidities between patients diagnosed with CAI or HAI in the current study.

It is noteworthy that patients with HAI had fewer symptoms and thoracic radiological alterations; however, they had more altered respiratory analytical parameters, more anemia and hypoalbuminemia and more complications leading to death. Other studies have reported a lower rate of pneumonia and respiratory failure and higher rates of mortality in patients with HAI.^5^, ^6^, ^11^ One explanation could be that CAI patients are admitted only for pneumonia, respiratory failure, or any criteria suggesting disease. Another surprising finding in this study was the low proportion of patients who received oseltamivir treatment (43.7%) and the high proportion of patients who received antibiotic treatment (78.9%). In previous studies, oseltamivir treatment was administered in greater than 65% of patients with HAI,^10^ while a range of 66%^4^‐71%^24^ received antibiotics. In the hospital, particularly in the emergency department, clinical suspicion of influenza in adults coincides with the majority of cases with acute respiratory infection (ILI) of viral etiology. In most cases, only the detection of influenza viruses in a respiratory sample allows for establishment a confirmed diagnosis. In our hospital, 22.3% of patients with ILI who attended the emergency department also had influenza; this rate is 20.4% and 23.5% in the pediatric and adult populations globally, respectively.^25^ Delayed diagnosis and the low proportion of patients with pneumonia or severe influenza, which were the criteria used up until 2018 in our hospital for the administration of oseltamivir, may explain the low use of oseltamivir. In our study, the delay between hospital admission and symptoms onset was 10 days, compared with 72 hours (48‐196) in 29 studies reviewed recently.^26^

The use of rapid diagnostic testing that allows a shorter turnaround time to receive the results might aid in the earlier initiation of oseltamivir treatment; unfortunately, such tests were not used in our institution. Additionally, this might allow for a reduction in the use of antibiotics and allow for more rapid initiation of preventive measures.^25^

There were no significant differences in the frequency of influenza subtypes A and B among those with CAI and HAI. These findings differ from those previously reported,^4^ implying that the risk of transmission is in hospitals and thus precautions need to be similarly implemented. Hemagglutinin, neuraminidase gene sequencing, or whole ‐ genome sequencing of the influenza virus have been suggested for detecting nosocomial influenza outbreaks more quickly and accurately; however, we did not have the means to implement these set tests in this study.^27^

This study has several limitations. There was a risk of loss of information regarding the prognosis of patients who were transferred to another hospital from the emergency department. Additionally, we did not have full access to the medical charts for patients who were previously admitted to other hospitals. Moreover the number of patients included was limited, and there might have been some differences in baseline pathologies or previous surgeries that were not evaluated in this study. These limitations have an impact on the validity and generalizability of our findings.

## 5 CONCLUSIONS

Hospital‐acquired influenza is common and it is associated with a greater frequency of complications and death. A significant proportion of CAI patients are admitted to the hospital and do not receive treatment or early isolation. The early diagnosis and treatment, and implementation of microbiological measures, will help to reduce the complications. Also, the implementation of preventive measures is the tools that will help to reduce the frequency of nosocomial transmission.

## CONFLICT OF INTEREST

The authors declare that there is no conflict of interest.

## AUTHOR CONTRIBUTIONS

**Maria Isabel Fullana Barceló:** Data curation (lead); Investigation (lead); Writing‐original draft (lead); Writing‐review & editing (lead). **Javier Asensio Rodriguez:** Data curation (equal); Investigation (equal). **Francisca Artigues:** Data curation (equal); Investigation (equal); Writing‐review & editing (equal). **Adrian Ferre:** Data curation (equal); Investigation (equal). **Pilar Salva:** Investigation (supporting). **Maria Almodovar:** Data curation (supporting); Investigation (supporting). **Pilar Sanchis:** Formal analysis (lead); Methodology (lead); Writing‐review & editing (supporting). **Maria del Carmen Lopez Bilbao:** Supervision (supporting); Validation (supporting); Writing‐original draft (supporting). **Jorge Reina:** Supervision (equal); Validation (equal); Writing‐original draft (supporting); Writing‐review & editing (supporting). **Melchor Riera Jaume:** Investigation (supporting); Methodology (supporting); Supervision (lead); Validation (lead); Writing‐original draft (supporting); Writing‐review & editing (supporting).

## ETHICAL APPROVAL

As an observational study, no intervention was performed in patients and no written informed consent was given to the patients. Medical charts and information were anonymized before the analysis.

### PEER REVIEW

The peer review history for this article is available at https://publons.com/publon/10.1111/irv.12823.

## Data Availability

The data that support the findings of this study are available from the corresponding author upon reasonable request.
